# Longitudinal pharmacogenomic analysis of refractory lung cancer to identify therapeutic candidates for epidermal growth factor receptor–tyrosine kinase inhibitor resistance subclones

**DOI:** 10.1038/s12276-025-01493-2

**Published:** 2025-07-04

**Authors:** Namhee Yu, Mihwa Hwang, Beung Chul Ahn, Youngjoo Lee, Sehwa Hong, Hanna Sim, Bo Ram Song, Sunshin Kim, Charny Park, Ji-Youn Han

**Affiliations:** Research Institute, National Cancer Center, Goyang-si, Republic of Korea

**Keywords:** Translational research, Cancer therapy

## Abstract

The dynamic nature of longitudinal tumor evolution across patients presents challenges in designing effective drugs. Here we aimed to determine tumor evolution resistance mechanisms and explore candidate drugs for specific tumor evolution types. We conducted longitudinal pharmacogenomic analysis of datasets of 73 samples in 34 patients among a National Cancer Center refractory lung cancer cohort (*n* = 199). Genomic profiles were determined to identify evolutionary trees in each patient, which were classified into tumor evolution groups according to the predominant truncal mutations, *TP53* and epidermal growth factor receptor. These groups were categorized into persistence, extinction and expansion groups according to the status of these two clones. Pharmacogenomic profile analysis identified that XAV-939 was effective for the epidermal growth factor receptor-extinction group exhibiting epithelial-to-mesenchymal transition-activated resistance. In addition, *MYC*^+^ subclones were maintained similarly to drug-tolerant residual cells throughout the evolution period. Moreover, *MYC*^+^ lung adenocarcinoma showed a poor outcome and had higher risk of transformation to small-cell lung cancer. Furthermore, the epithelial-to-mesenchymal transition-activated and *MYC*^+^ subclones were implicated in concurrent epidermal growth factor receptor–tyrosine kinase inhibitor resistance. Finally, our drug screening identified barasertib, an aurora kinase inhibitor, as a triple-combination candidate with epidermal growth factor receptor–tyrosine kinase inhibitors and XAV-939 for *MYC*^*+*^ cells. This study demonstrates the utility of longitudinal pharmacogenomic analysis to develop treatment strategies according to individual tumor evolution type. The study underscores the importance of integrating genomic and pharmacogenomic profiling in clinical practice to tailor treatments according to tumor evolution type.

## Tailored treatments emerge from tumor evolution insights

This study investigates the evolution of lung cancer and its resistance to treatment, focusing on the mechanisms driving cancer cell survival under therapy and their evolution over time. To explore these processes, patient-derived cells were utilized. Patient-derived cells are cells collected from patients and cultured in the laboratory, providing an opportunity to test various drug treatments. They used advanced techniques, including next-generation sequencing and single-cell transcriptome analysis, to track how cancer cells evolve. They identified different types of tumor evolution (persistence, extinction and expansion) and found that certain drugs could target specific cancer cell types that emerge during treatment. The authors concluded that understanding these evolutionary patterns can facilitate the development of better treatments tailored to individual patients.

This summary was initially drafted using artificial intelligence, then revised and fact-checked by the author.

## Introduction

Targeted therapies show promising initial responses in non-small-cell lung cancer (NSCLC) with actionable genetic alterations; however, these responses are often incomplete and temporary^1^. Therapy-induced tumor evolution leads to genetic heterogeneity, with differences emerging between the primary tumor and metastasis and resulting in multiregional genetic propensity within a tumor over time^2^. Tumor evolution exhibits distinct patterns of clonal selection, which may be extinction, persistence or acquisition^3^. Convergent evolution can cause truncations in evolution trees^4^. These evolutions exhibit dynamic heterogeneity across tumor clones and tumor-associated microenvironments. Drug-tolerant cells clearly differ from those constituting primary tumors, but residual cells tend to maintain cancer stem cell transcriptome in a steady state. Meanwhile, the persistent clone expansion remains unknown, making it difficult to distinguish the acquired evolving subclones of resistance^5^. Resistance mechanisms and clonal resistance of NSCLC are well known; however, the fate of residual cells is not fully understood^1^. Furthermore, despite identifying subclones and their therapeutic variants, identifying effective drugs on the basis of clonal markers remains challenging.

Epidermal growth factor receptor (EGFR)–tyrosine kinase inhibitor (TKI) resistance induces widespread changes across mutation profiles, cell populations and tumor-associated microenvironments^6,7^. Moreover, histological transformation to small-cell lung cancer (SCLC) can lead to drastic refractory characteristics out of precision medicine^8^. Although evolutionary alterations have been well identified from longitudinal tracing of genomic profile, resistance mechanisms are known to restrict drastic clonal changes. Moreover, therapeutic target variants do not ensure better efficacy and responses for progressed tumors^9^. Furthermore, regarding genomic profiles, phenotypic evidence, such as drug response, can potentiate explorations of patient-tailored treatments and uncover response mechanisms^10^.

We have already established a pharmacogenomic platform using patient-derived cancer cells (PDCs) of refractory lung cancer^10^. Multi-omics profile of our PDCs already demonstrated the concordance with clinical profile of pathologic subtype, overall survival, smoking and EGFR–TKI treatment progression. PDCs could be effectively used to investigate patient-tailored drug candidates^11,12^. PDCs are obtained from liquid biopsies, making them suitable for early detection, real-time monitoring of treatment status and rapid evolution, unlike tumor tissue biopsies. Our platform enables the rapid exploration of drug candidates and resistance mechanisms by closely monitoring patients with refractory lung cancer.

Therefore, this study aimed to leverage our pharmacogenomic platform to investigate drugs for longitudinal tumor progression. Tumor evolution types were classified using genomic profiles. Sensitive drugs for each evolution type were explored. Candidate treatments and mechanisms were ultimately demonstrated using single-cell profiles and external lung cancer cohorts.

## Materials and methods

### Longitudinal pharmacogenomic profile of PDCs derived from a refractory lung cancer patient cohort

PDC samples along with clinical profiles were obtained from patients with advanced or refractory lung cancer at the National Cancer Center (NCC) in the Republic of Korea^10^ (Supplementary Table 1). Multiple classes of drugs (*n* = 48) were used for drug response screening (Supplementary Table 2). All screening compounds were purchased from Selleckchem. This study was approved by the institutional review board of the NCC (Goyang, Korea; protocol number NCC2016-0208). All patients provided written informed consent.

### Next-generation sequencing data processing

Our targeted-sequencing and RNA sequencing (RNA-seq) pipeline were based mainly on the Genomic Data Commons pipeline used in our previous study^10^. In addition, somatic mutations were identified using Mutect2 and annotated by Oncotator^13^. Copy number variants were identified using GATK4.0.4.0, and peak calling was performed using GISTIC2^14^. Bad-quality reads were trimmed and aligned to the reference human genome GRCh38 using the STAR two-pass method for gene expression profile analysis^15,16^. Gene expression profiles were estimated using RSEM^17^. Fusion genes were called and summed from STAR-fusion and PRADA results^16,18^. Fusion genes were annotated using Pegasus^19^. Gene set enrichment analysis (GSEA) using differentially expressed genes (DEGs) was performed for several gene sets by referring to Gene Ontology, Kyoto Encyclopedia of Genes and Genomes, HALLMARK and WikiPath using the R package fgsea version 1.28^20^. Scoring of pathway or gene signature gene sets was performed using Gene Set Variation Analysis (GSVA) version 1.46^21^.

### Tumor evolution type classification in each patient using mutation profile

We used TreeOmics version 1.9.2 to identify metastatic seeding patterns in each patient to dissect subclones and their phylogenies from longitudinal bulk samples. Mutation read count and coverage tables with options were input (-e 0.01, -z 0.5)^22^, and the ratio of nonsynonymous to synonymous mutations (dN/dS ratio) was calculated from the mutation profiles using dNdScv^23^. The MATH score was calculated as follows: MATH_*i*_ = median absolute deviation (MAD) × variant allele fractions (VAF_*i*_)/median (VAF_*i*_) × 100 for sample *i*. In addition, we collected lung cancer mutation profiles to extract mutation genes increasing in metastasis^24^. Odds ratios were determined, and chi-squared tests were performed for each contingency table derived from mutation and metastasis statuses.

### Longitudinal drug response analysis and DNN to infer drug gene signature

We performed paired t-test to obtain drug area under the curve (AUC) values for the *EGFR* and *TP53* evolution groups. In addition, we compared the evolution types and responses at time point 1 (*T*_1_) with those at time point 2 (*T*_2_) within the evolution type to assess the response difference. Finally, candidate drugs were identified using a cutoff of *P* < 0.1 and log_2_ fold change (FC) >0.2.

Subsequently, we established deep neural network (DNN) models to predict drug response (AUC values) from input transcriptome profiles to identify drug-associated regulatory mechanisms. The pharmacogenomic dataset for DNN was collected from a NCC PDC cohort (*n* = 199). DNNs were applied using the H_2_O package. DNN model hyperparameters (hidden layer, regularization parameters, epoch, dropout ratio and nfolds) were optimized using the grid search method (Supplementary Table 3). Next, we selected the best-performance models by grid search to have the smallest root mean squared error value for each drug. Finally, a drug gene signature for each drug was extracted from the top-ranked genes (*n* = 100) by DNN feature importance to indicate the relative importance of learning model contribution. Pathways for each drug gene signature were inferred with GSEA using fgsea^20^.

### Single-cell transcriptome to dissect therapeutic subclones and resistant cells

The H1975 (EGFR-L858R/T790M) cell line, obtained from American Type Culture Collection, was cultured in Roswell Park Memorial Institute medium (1640, #10-040-CVRC; Corning) supplemented with 10% fetal bovine serum (#35-015-CV; Corning) at 37 °C in a humidified atmosphere with 5% CO_2_. Cells were seeded at 1 × 10^6^ on a 100-mm dish for each sample. After overnight incubation, cells were treated with 0.02 μM AZD9291 (Selleckchem) or 50 μM XAV-939 (Selleckchem) or a combination of AZD9291 + XAV-939 or dimethyl sulfoxide as the control. After incubation for 72 h, cells were collected for single-cell transcriptome analysis.

Samples were labeled using a BD single-cell multiplexing kit (BD Biosciences) and subjected to cDNA library construction using the BD Rhapsody system, BD Rhapsody WTA amplification kit and BD Rhapsody cDNA kit (BD Biosciences), to acquire single-cell transcriptome profiles. Paired-end sequencing was performed using the Illumina HiSeq 2500 sequencer (Illumina). Subsequently, single-cell-level gene expression profile analysis was performed using the BD Rhapsody Analysis pipelines. Each cell was filtered out to be double tagged by BD Rhapsody, mitochondrial percent (>30%) and feature count (<200 or >6,000) in the quality check process. Finally, the unique molecular identifier (UMI) count was acquired as the gene expression profile. Moreover, we performed sample merging, normalization, rescale and dimension reduction (Uniform Manifold Approximation and Projection, UMAP) by Seurat^25^. Clustering and cell cycle scoring were performed, and DEGs were sequentially examined for predefined clusters. A trajectory was inferred, and its pseudo-time was extracted. Finally, we identified two drug-tolerant cell populations based on therapeutic response, termed resistant and residual clusters. Drug-tolerant gene signatures for these cell populations were extracted from the top-ranked DEG set. The pathway scores were assessed using Seurat, with reference to the HALLMARK pathway.

### Drug combination response evaluation

H1975 cells were seeded in 384-well plates (1,000 cells per well) in quadruplicate for each treatment. After overnight incubation, cells were treated with drugs at the indicated concentrations for each drug (XAV-939 and repotrectinib) combined with AZD9291. Cell viability was measured after 72 h of treatment using the CellTiter-Glo Luminescent Cell Viability Assay kit (Promega, cat. no. G7573). The synergy scores of the expected drug combination responses were calculated on the basis of the highest single agent (HSA) model using the SynergyFinder 3.0 Web application. The combination index (CI) was calculated using the CalcuSyn software (Biosoft) based on the multiple drug-effect equation of Chou–Talalay.

### Drug-tolerant subclone evaluation from multiple cohorts

We collected bulk transcriptome datasets from Cancer Genome Atlas Lung Adenocarcinoma (TCGA LUAD), Gene Expression Omnibus (GEO) NSCLC and our NCC PDC cohorts to evaluate drug-tolerant cells. Simultaneously, we prepared gene signatures for each therapy-induced cell type. We manually determined 15 pathways (Supplementary Table 4) and calculated activity scores using GSVA to assess the activation status of TKI-resistant mechanisms^21^.

Furthermore, we collected transcriptome profiles from TRACERx and SCLC transformation to investigate cell populations in metastasis and histological transformation^26,27^. In TRACERx profiles, we assessed the difference in cell type scores between primary–primary (P–P) and primary–metastasis (P–M) in each patient. In SCLC transformation profiles, we assessed cell type scores for each sample. Moreover, cell score differences between the two groups were assessed using a customized *t*-test. Cox hazards survival analysis was performed using survival version 3.5 R package, resulting in two groups based on drug-tolerant scores: high (upper quartile) and low (lower quartile). These drug-tolerant cell fractions were identified from the bulk transcriptome datasets using cell deconvolution package MuSiC version 1.0^28^.

### Drug-tolerant cell types to predict therapeutic resistance and prognosis

We assessed the drug-tolerant cell population status of drug-tolerant clones among EGFR–TKI-treated patients. The clinical profile of patients receiving EGFR–TKI was collected from the NCC PDC cohort. We categorized the patients into four groups according to sequential treatment: BASELINE, treatment-naive patients; POST1, those who received first- or second-generation EGFR–TKI; POST2, those who acquired resistant mutations (EGFR.T790M) after EGFR–TKI treatment; and POST3, those treated with AZD-9291 (osimertinib). Drug-tolerant cell fractions were calculated via MuSiC version 1.0 using the transcriptome profile of these treated patients. Next, we assessed AUC scores using pROC version 1.18.5 to indicate prediction performance of patients’ drug-tolerant cell factions for each treatment group.

Before the survival test, we grouped the NCC PDC cohort into two subgroups based on the score for each cell type: low (lower quartile) and high (upper quartile). A 5-year survival test was performed to assess the overall survival between the low and high score groups using a Cox hazards regression model. In addition, seven NSCLC transcriptome profiles were collected from the GEO and TCGA datasets (GSE41271, GSE68465, GSE11969, GSE13213, GSE120622, GSE31210 and TCGA LUAD) to perform a cross-evaluation of the survival analysis.

## Results

### Evolutional model classification inferred from longitudinal variant profile of refractory lung cancer

PDC samples were collected from an NCC refractory lung cancer cohort (*n* = 199). In total, 73 longitudinal samples were obtained from 34 patients across 2–4 time points of therapeutic progression. Both NSCLC (*n* = 69) and SCLC (*n* = 6) cases were included, and histological transformation from LUAD to SCLC was not observed in any case. Genomic profiles were extracted from targeted sequencing and RNA-seq, and drug response screening was simultaneously performed using 48 anticancer compounds targeting angiogenesis (*n* = 3), cell cycle (*n* = 8), DNA damage (*n* = 5), MAPK (*n* = 3), PI3K–AKT–mTOR (*n* = 3), protein tyrosine kinases (PTK, *n* = 7) and others (*n* = 14). The PDC samples were mostly obtained from liquid biopsies: pleural effusion (93%), pericardial effusion (3.5%), ascites (1%) and tissue (2.5%). PDC samples were previously demonstrated to show highly correlated malignancy with the transcriptome profiles of LUAD tissue biopsies^10^.

**Fig. 1:**
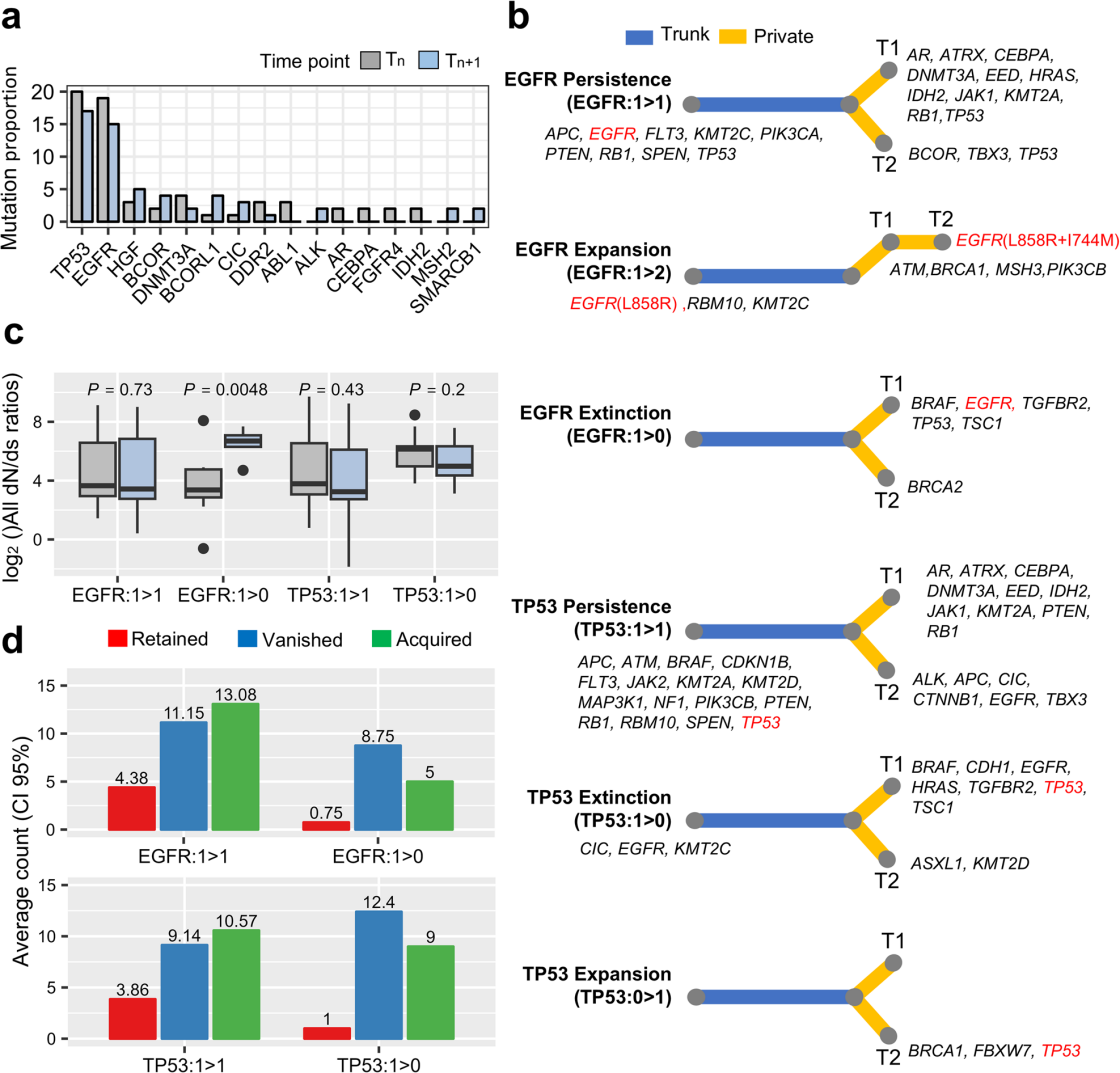
The genomic profile to categorize therapy-induced evolution models. **a** The mutation frequency of longitudinal refractory lung cancer at each time point (early *T*_*n*_, late *T*_*n*+1_). **b** Therapy-induced evolution models clustered according to recurrent mutation *TP53* and EGFR. Mutations corresponding trunk and branch (private) are marked. **c** Ratios of nonsynonymous-to-synonymous mutations to infer positive or negative selection of evolution models. **d** The average count of fusion genes of the evolution models. Retained, vanished and acquired variants are separately counted at time points *T*_*n*_ and *T*_*n*+1_.

When examining the genomic profile of longitudinal cases, a median of five mutations on the exon target regions of 386 genes were identified per sample. *TP53* and *EGFR* presented most recurrent mutations, and their frequencies slightly decreased at later time points (Fig. 1a). We assessed the tumor evolution trees in each patient using variant profiles, to categorize the truncal and private variants for time points (Supplementary Table 5). *EGFR* and *TP53* were the top truncal favored mutated genes compared with other genes. Therefore, their evolution trees were clustered on the basis of these two recurrent truncal mutations^3^ (Fig. 1b). The first observation time was defined as *T*_*n*_, and the next time point was *T*_*n*+1_ for each patient. Based on tumor progression from *T*_*n*_, we classified the evolution model into three types based on the presence of these major truncal variants: (1) persistent, which retained the truncal variants or tumor clonality across two time points (*T*_*n*_ and *T*_*n+*1_); (2) extinction, wherein variants disappeared in *T*_*n*+1_; and (3) expansion, wherein variants or clonality were acquired in *T*_*n*+1_ (ref. ^3^). Accordingly, patients with *EGFR* and *TP53* mutations were divided into the EGFR persistence (EGFR: 1 > 1, *n* = 13), extinction (EGFR: 1 > 0, *n* = 4) and expansion (EGFR: 1 > 2, *n* = 1) groups and *TP53* persistence (TP53: 1 > 1, *n* = 14), extinction (TP53: 1 > 0, *n* = 5) and expansion (TP53: 0 > 1, *n* = 3) groups, respectively. The evolution trees of SCLC (n = 3) were also investigated. Although SCLC progression shared *TP53* and *RB1* in the truncal or *T*_*n*+1_ private branch, it was inadequate to classify SCLC into more detailed subgroups.

Three evolution models exhibited distinct variant burden with progression. We calculated the adjusted dN/dS ratio to determine positive and negative selections^26^. Changes in this ratio revealed the different trends among evolutionary groups. The dN/dS ratio of the EGFR-extinction group (EGFR: 1 > 0; *P* = 0.0015) substantially increased compared with that of the TP53 extinction group (*P* = 0.049; Fig. 1c). However, no notable change was observed in the dN/dS ratio in the persistence groups, indicating that the progression after treatment with EGFR–TKI facilitates positive selection of somatic mutation. We categorized the fusion genes into three fusion types: (1) maintained type, which existed all time points; (2) vanished type, which did not exist at late time point; and (3) acquired type, which newly observed at late time points (Fig. 1d). Persistence models of both *EGFR* and *TP53* contained twice the number of vanished or acquired fusions compared with the retained type (Fig. 1d). Although the fusions of extinction models mostly vanished, acquired fusions were relatively lower. However, the expansion evolution models exhibited no concordance between the *EGFR* and *TP53* models owing to a limited number of expansion model cases, which may have resulted in inconclusive results. In summary, the EGFR extinction of somatic cells consequently elevated positive selection in progression, whereas it reduced fusion gene burden.

In addition, the EGFR-expansion model emerged as mutation positive based on therapeutic evolution, except for that in one patient who harbored EGFR.L858R and acquired a secondary point mutation EGFR.I744M after EGFR–TKI treatment (Fig. 1b). Second mutations were extensively identified in 25% of patients among 288 EGFR-mutated profiles in a metastatic NSCLC cohort of MSK (*n* = 2,621)^29^. Among these second mutations, T790M and additional rare second mutations were revealed to be highly selective to AZD9291 (ref. ^30^). However, EGFR.I744M was not detected in the MSK cohort and was previously not reported to demonstrate TKI responses. Our PDC drug screening system successfully determined whether EGFR.I744M was TKI-sensitive or not. The patient indicated the best sensitivity to three EGFR–TKIs at early time point *T*_*n*_ but acquired resistance due to the second mutation for all three TKIs (Supplementary Fig. 1). Consequently, EGFR.I744 promotes resistance, unlike the T790M second mutation. Thus, our screening system can predict patient-tailored drug susceptibility of unexpected novel variants without time-consuming experimental validation. Our PDC platform successfully classified therapeutic evolution models, and the EGFR–TKI drug screening results showed significant concordance with patients’ treatment profiles.

### Pharmacogenomic analysis by longitudinal progression of evolution models

**Fig. 2:**
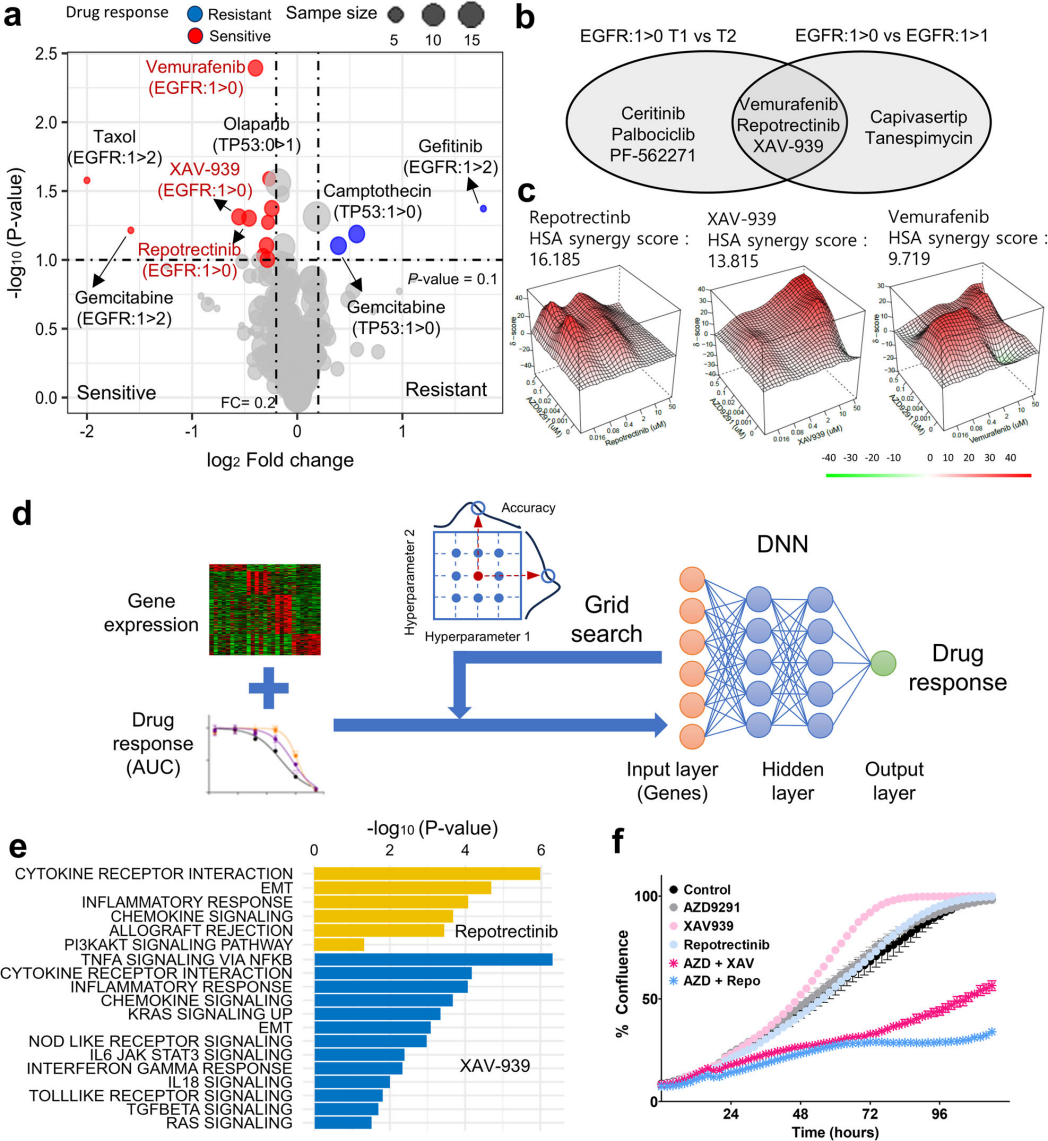
Pharmacogenomic analysis to investigate therapeutic candidates and mechanisms for evolution models. **a** A volcano plot to test drug response differences for each model. The *x* axis presents the log_2_FC of AUC values of *T*_*n*_ and *T*_*n*+1_. The *y* axis presents the log-scale *P* value by the Wilcoxon-rank sum test. Dot sizes indicate the number of samples. Cutoffs (log_2_FC of 0.2 and *P* = 0.1) are guided by dotted lines. **b** A Venn diagram of sensitive drugs of EGFR-extinction and EGFR-persistence groups. Drug candidates for two EGFR groups are extracted to be sensitive in T_*n*+1_. **c** The highest single-agent synergy scores of EGFR–TKI for three candidates. **d** DNN establishment to uncover therapeutic mechanism. Learning model using gene expression predicted drug responses, and the model is optimized by grid search. **e** The therapeutic mechanism delineated by drug gene signature. Pathways are detected by GSEA for repotrectinib and XAV-939. The *x* axis indicates the GSEA *P* values, and the *y* axis indicates the tested pathways. **f** Cell proliferation assay for six conditions by IncuCyte instrument: control, AZD9291 (0.02 μM), XAV-939 (50 μM), repotrectinib (0.4 μM), AZD9291–XAV-939 combination (AZD + XAV) and AZD9291–repotrectinib combination (AZD + Repo). Representative plots from three independent experiments are shown. Confluence was measured in triplicated wells (from 4 fields each). A Student’s *t*-test was performed for AZD9291 versus AZD + XAV, AZD9291 versus AZD + Repo, XAV-939 versus AZD + XAV, and repotrectinib versus AZD + Repo. All *P* values are less than 0.0001.

To overcome resistance through patient-specific therapeutic evolution, we investigated drug candidates on the basis of longitudinal evolution models. Sensitive drugs were tested for *T*_*n*+1_ samples comparing with paired *T*_*n*_ for each longitudinal evolution type (Figs. 2a, b; *P* < 0.1, |log_2_FC| >0.2). The *TP53* extinction model acquired resistance to camptothecin (DNA damage) and gemcitabine (DNA damage). The *TP53* evolution models were not sensitive to any drugs, whereas the EGFR-extinction model was sensitive to three molecules: vemurafenib (a BRAF inhibitor), XAV-939 (a Wnt inhibitor) and repotrectinib (a multi-TKI). When assessing synergy scores to determine the potential of EGFR–TKI combination therapy, repotrectinib and XAV-9393 exhibited better TKI combination effects than vemurafenib (HSA synergy score for vemurafenib 9.719, for repotrectinib 16.185 and for XAV-939 13.815; Fig. 2c). However, a drastic drug response change at *T*_*n*+1_ was not observed, because the EGFR-persistence group experienced an ongoing progression to therapeutic evolution with the current TKI treatment. Conversely, the EGFR-extinction group terminating TKI showed notable sensitive drugs compared with the other groups. Conversely, patients with the *TP53* evolution model not receiving targeted therapies exhibited divergent responses to sensitive drugs, with some reaching the significance cutoff.

Large-scale transcriptome profile can elucidate biological mechanisms associated with each drug response^12^. Therefore, we further investigated drug-associated gene signatures and pathways for sensitive drug candidates (Fig. 2d). To enhance statistical performance, we established DNN models for each drug using both transcriptome and drug screening profiles acquired from all NCC PDC cohort samples (*n* = 199). Learning model hyperparameter optimization was performed by grid search for the best accuracy, root mean squared error (Supplementary Table 3). We successfully established the best performance learning model for each drug and ranked genes by the importance feature for each drug. Finally, we determined the drug gene signatures of the top-ranked 500 genes.

Drug signatures obtained from DNN delineated target regulatory mechanisms. Repotrectinib and XAV-939, targeting the EGFR-extinction group, concurrently targeted cytokine receptor interaction, Epithelial to mesenchymal transition (EMT) and chemokine signaling (*P* < 0.1; Fig. 2e). In addition, XAV-939 was susceptible to tumor necrosis factor (TNF)-alpha and tumor growth factor (TGF)-beta signals. This implies that EMT and chemokine signaling facilitated by EGFR–TKI is inhibited by XAV-939 or repotrectinib. In terms of TKI resistance prevention, XAV-939 and repotrectinib showed a good response when combined with EGFR–TKI. Therefore, we additionally screened responses for single and combination treatment cases (Fig. 2f). The single treatment condition involved AZD9291 treatment, whereas combination therapy was tested with repotrectinib and XAV-939. As expected, combination treatment showed better performance than monotherapy (Fig. 2f). In particular, XAV-939 showed a better combination effect than repotrectinib. Target mechanisms of EGFR–TKI resistance were identified using a machine-learning approach; however, therapy-induced subclones were not precisely characterized. To understand TKI resistance at the clonal level and the target mechanism of the candidate drug XAV-939, we further analyzed single-cell transcriptome profiles from TKI resistance to combination therapy and assessed the subclonal status of patients using bulk tumor samples.

### Single-cell transcriptome to dissect therapy-induced cell population

**Fig. 3:**
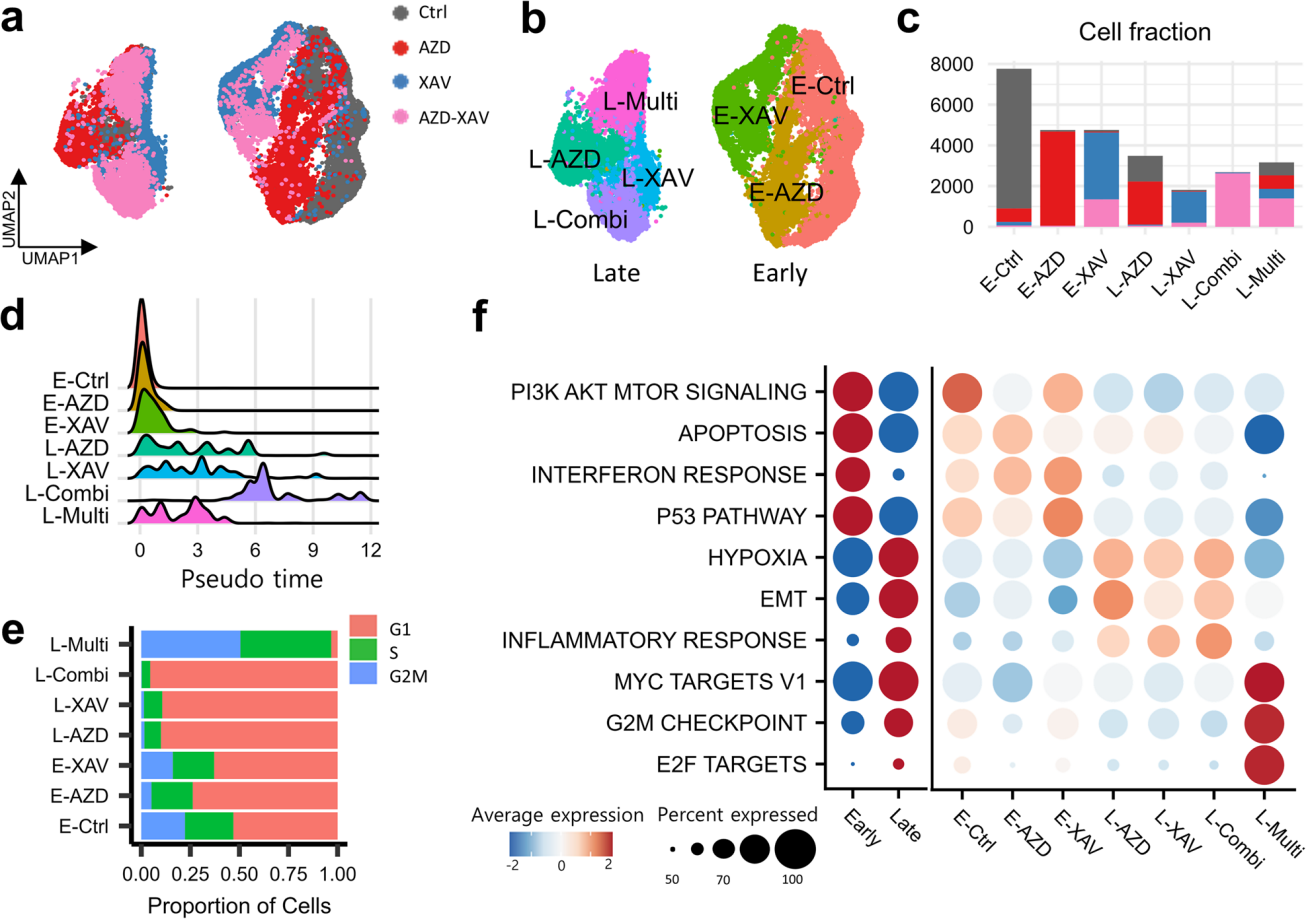
Therapy-induced subclone identification using single-cell transcriptome profiles. **a** A UMAP projected by single-cell transcriptome profiles of total cell models. Control (Ctrl) is the untreated H1975 cell line, AZD the AZD9291-treated model, XAV the XAV-939-treated model and AZD–XAV the combination therapy model of AZD9291 and XAV-939. **b** Single-cell clustering to identify seven therapeutic subclones. **c** The cell counts of treatment condition of the seven subclones. **d** Pseudo-time distributions for seven subclones to speculate therapeutic evolution time. Trajectory analysis by therapy is performed using Seurat. **e** Cell cycles G1, S, and G2M are inferred from single-cell transcriptomes for subclones. **f** Heatmaps of pathway scores according to single-cell clusters.

Although pharmacogenomic platform analysis effectively infers drug candidates, bulk sample analysis remains ambiguous in determining whether evolutionary resistant subclones are precisely targeted by drugs and which residual cells persist to drive final refractoriness. Therefore, we leveraged resistance cell models of monotherapies and combination treatment (AZD-9291 and XAV-939) and dissected therapeutic subclones from single-cell transcriptome profiles to assess the clonal heterogeneity by evolution models. We induced drug-tolerant cell models for monotherapy and combination therapy. The H1975 cell line having *EGFR* L858R/T790M mutations was selected. We optimized treatment time to 72 h for several conditions to retain residual cells after treatment^31^ (Fig. 2f). Subsequently, single-cell transcriptome profiles were generated using untreated control and AZD-9291 and XAV-939 treated cells, and quality control was performed. In total, 28,423 cells were identified, including untreated (31.615%), AZD-9291-treated (28.642%), XAV-939-treated (19.523%) and combination-therapy-treated (20.216%; Fig. 3a) cells.

Our drug-tolerant cell models were globally divided into two clusters according to the therapeutic status of early and late resistance (Fig. 3b). These cells were further clustered into seven subclusters based on experimental conditions: early-control resistance (E-Ctrl), early-AZD-9291 resistance (E-AZD), early-XAV-939 resistance (E-XAV), late-AZD-9291 resistance (L-AZD), late-XAV-939 resistance (L-XAV), late-combination therapy resistance (L-Combi) and late-multiple condition residual cell (L-Multi; Fig. 3b, c). Known canonical markers were detected in our analysis of DEGs for each single-cell cluster: *CAPS*, *KRT19*, *KRT8* and *MUC5B* in E-Ctrl-like epithelial cells; *CD44*, *COL12A1* and *TGFBR3* in L-Combi-like EMT; and *AIMP2* and *NME1* in L-Multi-like MYC-activated cells (Supplementary Table 6).

The subcluster regulatory program exhibited distinct cellular characteristics according to treatment. Three early resistance clusters in the trajectory analysis exhibited shorter pseudo-time distributions (Fig. 3d). L-AZD and L-XAV presented more progression and had higher pseudo-time than early clusters. L-Combi exhibited and extended final evolution evolving time induced by combination therapy. Conversely, L-Multi cells evenly originated from all experimental conditions and exhibited sustained cell proliferation across start to mid-time. Therefore, the L-Multi cluster was considered a residual cell type to maintain cell populations without therapeutic dependency (Fig. 3c). Six subclusters, except L-Multi, were present mostly in the G1 phase, increasing with therapeutic resistance acquisition (L-Combi); however, L-Multi was in the S–G2M phase, as the cells were proliferative (Fig. 3e). Next, the early resistance group exhibited activation of the PI3K–AKT–mTOR signaling pathway and the P53 pathway, both contributing to oncogenic signaling (Fig. 3f). The late resistance group showed the acquisition of EMT, MYC and G2M checkpoint disruption. At the subcluster level, late monotherapy (L-AZD and L-XAV) and combination therapy (L-Combi) clones shared hypoxia, EMT and inflammatory response activation by therapeutic evolution. However, only L-Multi exhibited the proliferative characteristics in response to MYC target, G2M checkpoint and E2F target. This implies that EGFR–TKI (AZD9291) treatment elevates EMT-activated subclones compared with E-Ctrl (L-AZD log_2_FC of 1.614). Consequently, XAV-939 (L-XAV) is a plausible candidate to target EMT inhibition for TKI resistance compared with AZD9291-driven EMT activity (L-XAV log_2_FC of 1.109, and L-Combi log_2_FC of 1.405). Furthermore, progressed therapy-resistance subclones converged to L-Combi. Thus, L-Multi is a persistent cell type that exists at low frequencies across all conditions; therefore, it appears to consist of refractory and residual cells distinguishable from other resistance subclones. Consequently, we hypothesized that drug tolerance could be categorized into two types: therapy-induced resistance and residual type.

### Therapy-induced subclones delineate tumor intraheterogeneity and progression

**Fig. 4:**
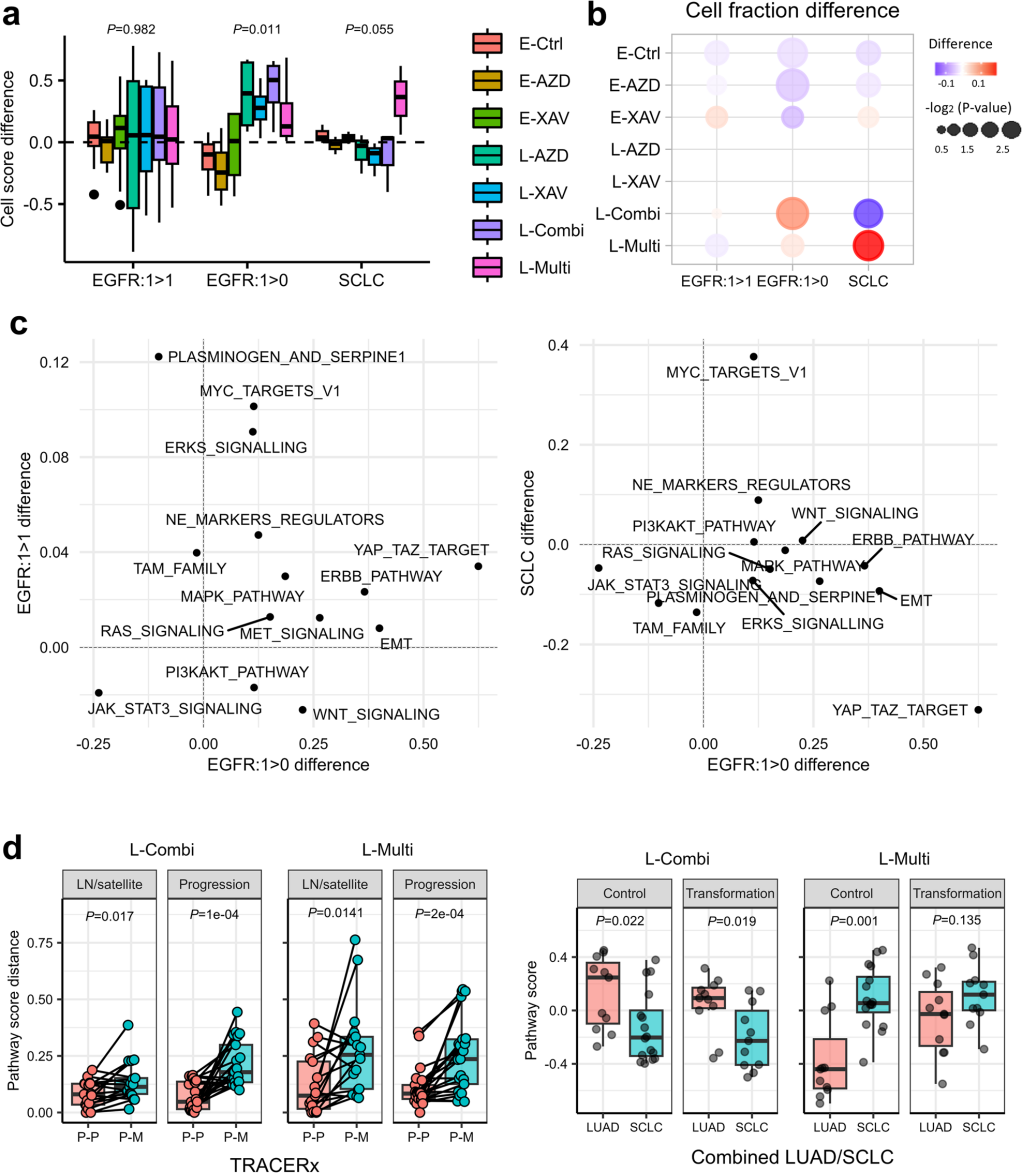
Therapy-induced cell subpopulation association with evolution models, regional heterogeneity and histological transformation. **a** A cell score bar plot of seven cell types for EGFR evolution models and SCLC. The *y* axis assesses the score difference between *T*_*n*+1_ and *T*_*n*_ scores within the evolution model. Each cell type is scored using GSVA. **b** A heatmap of *T*_*n*+1_ and *T*_*n*_ cell fraction difference for three groups: EGFR persistence, EGFR extinction and SCLC. **c** Two dot plots to compare TKI-resistant mechanisms’ activation status of the EGFR-extinction group with the EGFR-persistence and SCLC groups. **d** Box plots of L-Combi and L-Multi cell type scores in TRACERx and SCLC transformation transcriptome profiles. In TRACERx, P–P indicates the difference between primary pairs within a single patient, and P–M indicates the primary–metastasis comparison. Metastasis cases can also be categorized as being lymph node or satellite and as being recurrent or progressive. In SCLC transformation, control LUAD is never-transformed samples, and SCLC de novo. Paired samples are collected from SCLC cases transformed from LUAD. *P* values are acquired using the Wilcoxon-rank sum test.

We assessed changes in cell populations according to a progression in each evolution model using our patients’ transcriptome profiles to dissect longitudinal bulk samples into drug-tolerant subclones (Fig. 4a, b). The EGFR-persistence model (EGFR: 1 > 1) showed a difference between *T*_*n*+1_ and *T*_*n*_ without convergence, which was not significant compared with the population difference among the seven cell subclones (*P* = 0.982). Conversely, late drug-tolerant subclones in the EGFR-extinction model were clearly elevated in *T*_*n*+1_, and three early clones shrunk (*P* = 0.011). In addition, the abundance of L-Combi cells was mostly increased in *T*_*n*+1_, and L-Multi was relatively the lowest among all late drug-tolerant subclones. As our longitudinal samples included patients with SCLC, we further investigated the status of drug-tolerant subclones according to SCLC progression. L-Multi exhibited expansion with SCLC progression (*P* = 0.055), whereas the remaining six cluster types showed no difference. This implies that resistance subclones induced by EGFR–TKI and XAV-939 expand with TKI resistance and that EMT activation and cellular expansion are dominant after TKI treatment of NSCLC. By contrast, *MYC*^+^ drug-tolerant cells did not change with NSCLC progression but expanded with SCLC progression.

We further investigated the therapy-induced resistance mechanisms for each evolution model to uncover resistance mechanisms from transcriptomic evolution (Fig. 4c). EMT and YAP–TAZ regulations were activated with EGFR extinction, including the ERBB pathway (score difference >0.3). Moreover, transcriptomic change in the EGFR-persistence group was moderate. In addition, the progression within SCLC promoted MYC pathway activation, in contrast to YAP–TAZ downregulation.

We also evaluated the clonality status using additional transcriptome profiles of both TRACERx and SCLC transformation to assess whether therapy-induced subclones were associated with metastasis and transformation to SCLC^26,27^. TRACERx collected multiregional molecular profiles for each patient, including distant metastasis. First, we selected primary and metastasis samples for TRACERx (73 patients) and assessed the cell score difference between P–P (73 patients, 185 primary samples) and P–M (40 patients, 57 metastasis samples; Fig. 4d) samples. L-Combi and L-Multi cell populations were altered more in P–M than in P–P (L-Combi *P* = 1.9 × 10^−6^, L-Multi *P* = 4 × 10^−5^). Moreover, progressed tumors (P–M) exhibited stronger heterogeneity than lymph node or satellite metastasis (*P* = 2 × 10^−4^). When comparing between cell types, L-Multi showed a more drastic difference in both metastasis and progression compared with L-Combi. Taken together, Intratumor heterogeneity (ITH) of metastasis was relatively more dynamic than that of primary samples, and *MYC*^+^ proliferative cells could play residual refractory characteristics in tumor progression.

Next, we investigated our therapy-induced cell populations in SCLC transformation (Fig. 4d). We profiled paired samples of patients before and after SCLC transformation (*n* = 11), whereas control samples comprised de novo SCLC (*n* = 16) and never-transformed LUAD (*n* = 11) samples. L-Combi was more dominant in the LUAD group than in the SCLC group (control *P* = 0.022, transformation *P* = 0.019). However, L-Multi occupied substantial populations in SCLC (control *P* = 0.001, transformation *P* = 0.135), and transform-fate LUAD already harbored a substantial volume of L-Multi cells (*P* = 0.018). The driver genes and regulators of neuroendocrine SCLC have already been uncovered; however, genetic factors that determine transformation fate remain unknown^32^. Our results revealed that *MYC*^+^ LUADs were predisposed to transform to SCLC.

Consequently, drug-tolerant subclones were dichotomized into EMT-activated (L-Combi) and MYC^+^ (L-Multi) types. EMT-activated cells were mostly elevated, and YAP–TAZ upregulation concurrently facilitated the fibrotic cell reprogramming when acquiring resistance to EGFR–TKI. Meanwhile, *MYC*^+^ proliferative cell population moderately increased in EGFR–TKI resistance, but explosively expanded with SCLC transformation. Therefore, upregulated MYC expression in pretransformed LUAD could become a predictive marker of the progression to SCLC.

### Therapy-induced subclones predict therapeutic progression and survival

**Fig. 5:**
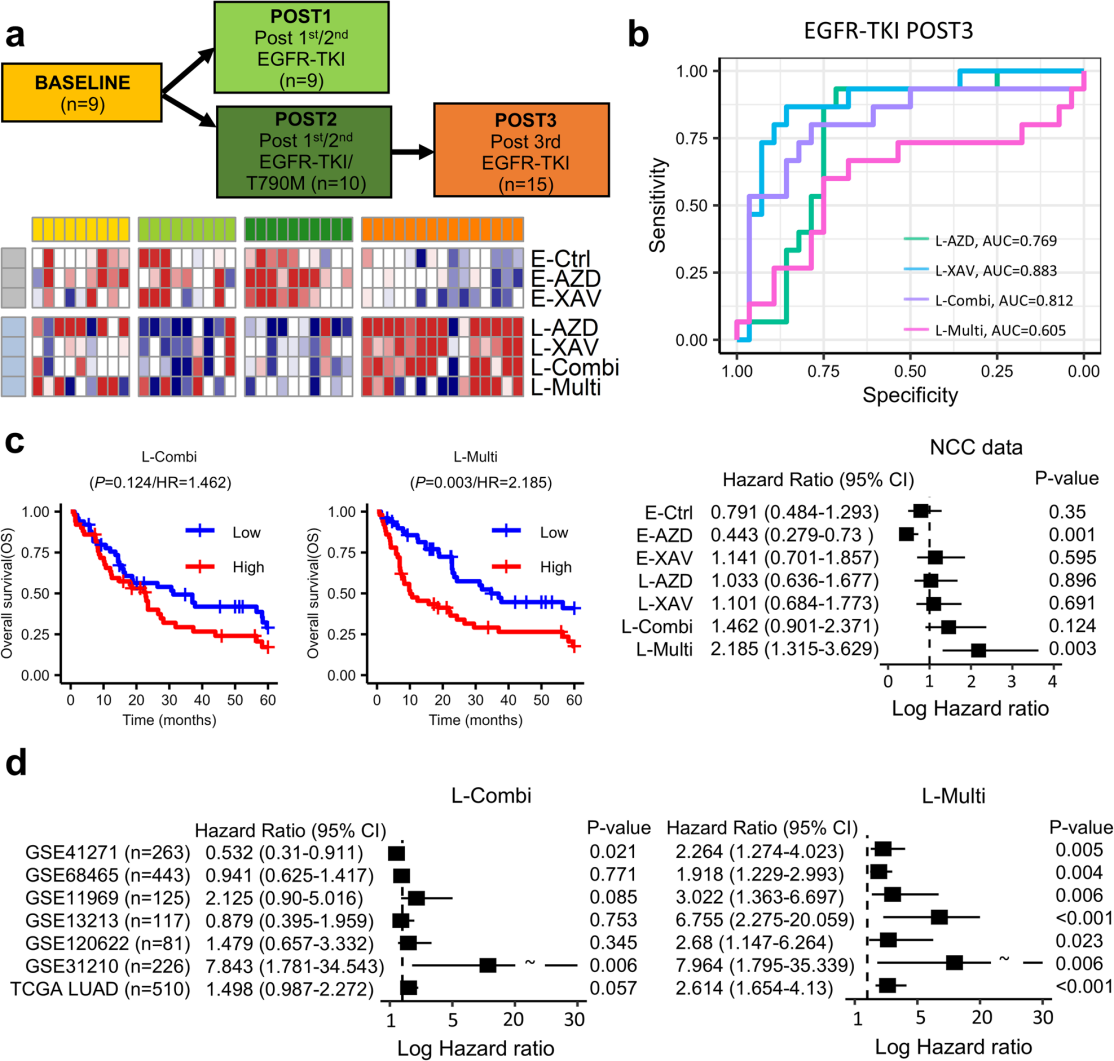
Therapy-induced cell types to predict the resistance and overall survival (OS). **a** A flowchart of EGFR–TKI treatment and its cell score heatmap using the NCC PDC cohort. **b** An AUC plot to investigate POST3 group prediction performance for each cell type. **c** Two survival plots for two resistant cell types: L-Combi and L-Multi. A Cox-proportional hazards regression model is used to assess the 5-year OS. Low and high cell scores are selected by lower and upper quartiles, respectively. A forest plot of 95% confidence interval log-scale HR (*x* axis) for seven cell types (*y* axis). **d** Two forest plots of HR derived from L-Combi and L-Multi cell type scores assessed from transcriptome profiles of seven NSCLC cohorts. *P* values are acquired by OS test. All methods and cutoffs are equal within the NCC PDC cohort.

We categorized patients from our PDC cohort according to step-wise EGFR–TKI treatment. In total, 43 patients were classified into four groups: BASELINE, treatment-naive (*n* = 9); POST1, resistant to first-line gefitinib or afatinib without T790M mutation (*n* = 10); POST2, resistant to first-line gefitinib or afatinib with acquisition of T790M mutation (*n* = 10); and POST3, those who developed resistance to osimertinib (*n* = 15; Fig. 5a). The scores of the seven drug-tolerant cell types were assessed from the transcriptome profiles of these patients. Three early-resistant cell types were activated across BASELINE to POST2, and four late-resistant cell types were dominantly abundant in POST3. Among them, L-XAV and L-Combi demonstrated the ability to predict (AUC >0.8; Fig. 5b) the final resistance group POST3, whereas L-Multi was less deterministic of POST3 (AUC 0.605). We performed a survival analysis of the NCC PDC cohort for the two strong drug-tolerant cell types, L-Combi and L-Multi (OS; Fig. 5c). The activation of both types predisposed poor prognosis. In particular, L-Multi dictated shorter survival time and worse prognosis than L-Combi (hazard ratio (HR) 2.185; *P* = 0.002). In addition, the HRs of L-Multi (HR median, 2.68) were consistently higher than those of L-Combi (HR median, 1.479) when evaluating survival across seven NSCLC cohorts using GEO transcriptomes (Fig. 5d). These results imply that EMT activation is involved in metastasis and facilitates progression, ultimately promoting poor prognosis. However, *MYC*^+^ regulation appears to be a stronger prognostic factor than the EMT pathway. Nonetheless, EMT activation is relatively favorable to determine the prognosis of EGFR–TKI resistance compared with *MYC*^+^.

### MYC-activated cells exhibited the sensitivity to cell cycle target drugs

**Fig. 6:**
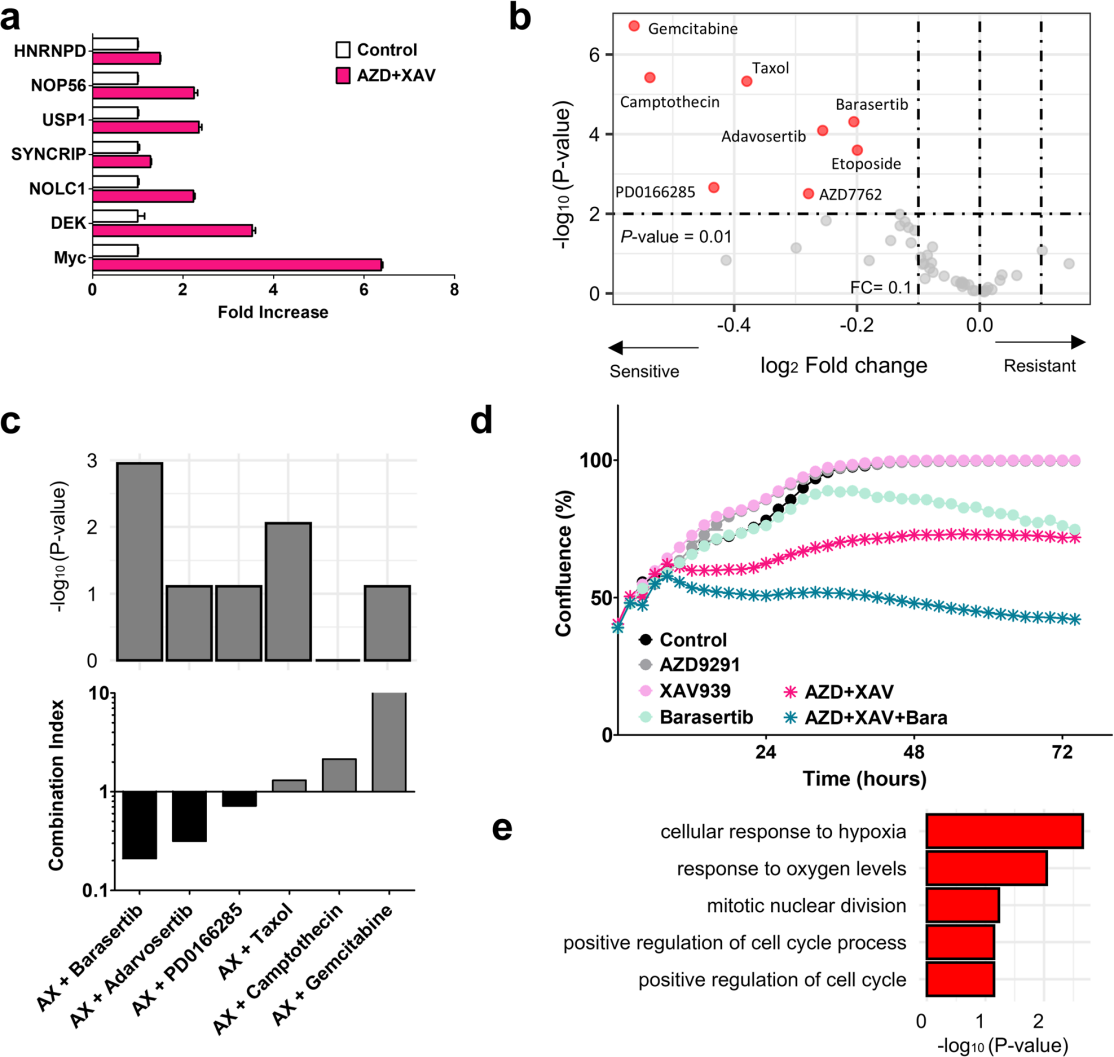
Therapeutic candidates for MYC^+^ cell target drugs. **a** A barplot of the mRNA expression of seven genes. Fold increases (*x* axis) in mRNA expression of each gene (*y* axis) are assessed using reverse-transcription polymerase chain reaction in H1975 cells treated with AZD9291 and XAV-939. **b** A volcano plot showing drug sensitivity differences between *MYC*-up and *MYC*-down groups. The *x* axis indicates drug FCs, and the *y* axis indicates the log-scale *P* value, determined using the Wilcoxon-rank sum test. **c** Top: barplot indicating enrichment log-scale *P* values between *MYC*^+^ upregulated DEG sets and DNN drug signatures. Bottom: barplot of drug CI for six drug candidates to combine with AZD9291 and XAV-939 (AX). **d** Cell proliferation assay for six conditions using the IncuCyte instrument: control, AZD9291 (0.02 μM), XAV-939 (50 μM), barasertib (10 μM), AZD9291–XAV-939 combination (AZD + XAV) and three-drug combination (AZD + XAV + Bara). **e** A barplot of *P* values of the *MYC*^+^ gene set pathway enrichment test in our PDC transcriptome profile.

We used TKI-resistant cell models and detected target drugs from our drug screening datasets to identify drugs for *MYC*^*+*^ residual cells to be tolerant to EGFR–TKI. The EMT and MYC-activated subclones were EGFR–TKI resistant. Therefore, the combination of AZD9291 and XAV-939 was suggested as the therapeutic strategy for EMT-activated cells. Subsequently, we investigated target drugs for the *MYC*^*+*^ residual cells to overcome EGFR–TKI resistance. We verified the overexpression of *MYC* and target genes by inducing a residual cell model using the H1975 cell line treated with a combination of AZD9291 and XAV-939 (Fig. 6a). We evaluated drugs that were sensitive to *MYC*^+^ cells using our pharmacogenomic dataset (Fig. 6b). Eight drugs were identified as significant, all of which shared common targets of DNA damage and cell cycle regulation. In addition, we assessed drug synergy using the CI (Fig. 6c). Cell cycle-targeting drugs barasertib and adavosertib exhibited better synergy than DNA damage-targeting drugs (Supplementary Fig. 2). We also assessed drug combination effects and DEG set enrichment of *MYC*^*+*^ samples with DNN drug response signatures. Cell cycle-targeting drugs exhibited better combination effects against resistant cells. Drug signatures were consistent with the significance in cell cycle drugs. The best candidate, barasertib, showed a drastic anticancer effect (Fig. 6d). The gene regulation by *MYC* was implicated in hypoxia and cell cycle (Fig. 6e). Barasertib-targeted AURKB upregulation was identified in *MYC*^*+*^ samples. As a result, TKI-induced resistance exhibited EMT activation, and these resistant subclones could be targeted by the XAV-939 combination. However, residual cells remained *MYC*^+^, suggesting that an AURKB cell cycle inhibitor could serve as an additional option to overcome the ultimate refractory nature of XAV-939 combination therapy.

## Discussion

Although longitudinal tracing has successfully categorized tumor evolution types, identifying underlying resistance mechanisms and effective drugs remains challenging. Moreover, the identification of therapeutic markers has not fully ensured the success of targeted therapies, as these treatments fail owing to the complexity of therapy-induced resistance and the heterogeneity of tumor evolution subclones^10,30^. Moreover, the evaluation of concurrent evolution features poses an additional challenge by personalized tumor evolution. In this context, our longitudinal pharmacogenomic profile simultaneously identified phenotypic drug and multi-omics response mechanisms according to therapy-induced evolution. Thus, we identified a candidate drug, XAV-939, to target EMT-activated subclones for EGFR-extinction evolution, which exhibited a better combination effect with EGFR–TKI than other compounds.

We categorized tumor evolution types into persistence and extinction groups on the basis of recurrent truncal mutations. *EGFR*-extinction groups targeted by TKIs exhibited significant DEGs and notable drug response changes with vanishing *EGFR* mutations. However, the persistence group showed no drastic changes in the pharmacogenomic profile. This implies that ongoing TKI treatment maintains the molecular mechanisms and drug responses of the tumors. Meanwhile, none of the *TP53* evolution groups exhibited drastic changes in their pharmacogenomic profile. This implies that targeted therapies, such as EGFR–TKI, induce predominant and conserved alterations in evolving tumors. Thus, the *TP53* mutation in NSCLCs is a predictor of a poor outcome^33^. However, patients with *TP53* mutations undergo sporadic evolution without targeted therapies. Therefore, tests for sensitive drugs and DEGs failed. To uncover evolutionary mechanisms for *TP53* mutation in lung cancer, more patient tracing cases should be collected for classifying more in-depth *TP53* mutation evolution groups. Furthermore, *TP53* co-occurrent variants, such as in *RB1*, *ARID1*, *KRAS* or *EGFR*, can specify mechanisms and dictate more treatment options, such as immunotherapy^34,35^.

The persistence or expansion subclone deconvolution by tumor evolution is a challenge in bulk transcriptome profile analysis. We leveraged single-cell transcriptome simulating therapy-induced evolution cell models and inferred corresponding cell populations from our bulk samples of longitudinal patients to address this issue. Our results distinguished both therapy-induced resistant cells and drug-tolerant residual cells arising from *EGFR* extinction. Moreover, LUAD transformation to SCLC involved a substantial amount of *MYC*^+^ residual cells. This highlights the importance of therapy development of *MYC*^+^ primary tumors to prevent SCLC transformation. The apolipoprotein B mRNA editing enzyme, catalytic polypeptide (APOBEC) signature and genome doubling time have already been identified as predictive indicators of histological transformation in lung cancer^8,36^. In addition, we demonstrated that *MYC*^+^ drug-tolerant residual cells promoted poor outcome compared with EMT-like features. The *MYC* gene regulates cell cycle activation, anti-apoptosis, metabolism modulation and the molecular characteristics of cancer stem cells^37^. Therefore, platinum-based drugs, such as cisplatin, are used for advanced disease in the standard treatment for NSCLC after first-line EGFR–TKI. However, *MYC*^*+*^ is involved in cisplatin resistance via induction of anti-apoptotic proteins blocking cisplatin-mediated death^38^. Moreover, MYC-dependent EMT facilitates invasion and metastasis in lung cancer^39^. Therefore, an alternative therapeutic strategy for *MYC*^*+*^ patients is necessary to prevent poor outcomes. Our pharmacogenomic profile suggests that cell cycle target drugs, including aurora kinase inhibitors, may target *MYC*^*+*^ cells, suggesting their potential utility to treat SCLC^40,41^. Therefore, instead of cisplatin, tailored treatments for *MYC*^+^ advanced lung cancer can improve clinical outcomes and prevent disease progression.

Furthermore, EMT activation induced by EGFR–TKI exhibited high concordance among patients with evolution. Therefore, pharmacogenomic analysis suggested a combinatorial therapeutic strategy to prevent TKI progression. Although our results are insufficient to verify EMT-activated subclones depending on stem-like cells, pharmacogenomic analysis supports the therapeutic candidates for EMT and *MYC*^+^ subclones. Thus, the identification of *MYC*^*+*^ can become an important factor to predict both clinical outcomes and proactive combination therapy substituting conventional platinum-based chemotherapy.

Our longitudinal pharmacogenomic analysis successfully identified subclones in EGFR–TKI-induced tumor progression and suggested therapeutic candidates for each subclone. *TP53*-dependent evolution pathways exhibit heterogeneous molecular characteristics, making it difficult to identify statistically significant regulatory mechanisms. Meanwhile, our analysis of SCLC transformation and TKI-resistant groups was still limited by the small sample size. To address this limitation, we conducted a comprehensive comparison with external cohorts, and our results demonstrated consistent clinical reproducibility. Sampling evolving SCLC and performing patient tumor biopsies during treatment present significant clinical challenges. Therefore, we anticipate that the accumulation of our PDCs will provide greater clinical relevance for future in-depth studies. Drug response prediction and genomic profiling for patient treatment monitoring remain an unmet clinical need. Our PDC platform demonstrated the potential to facilitate longitudinal resistance diagnosis and drug prediction for patients undergoing therapy in clinical settings.

## Supplementary information

Supplementary Information

Supplementary Tables

## Data Availability

Next-generation sequencing files of PDC samples from patients with refractory lung cancer have been deposited in the National Center for Biotechnology Information GEO (GSE165611 and GSE229535) and SRA (PRJNA694788).
