# Supplementary material for: Longitudinal pharmacogenomic analysis of refractory lung cancer to identify therapeutic candidates for epidermal growth factor receptor–tyrosine kinase inhibitor resistance subclones
Source: Exp Mol Med. 2025 Jul 4;57(7):1567–78. doi: 10.1038/s12276-025-01493-2 (PMC12322115; doi:10.1038/s12276-025-01493-2)
Supplement: Supplementary file 1 — Supplementary Information [file 12276_2025_1493_MOESM1_ESM.pdf]

Supplementary Figure

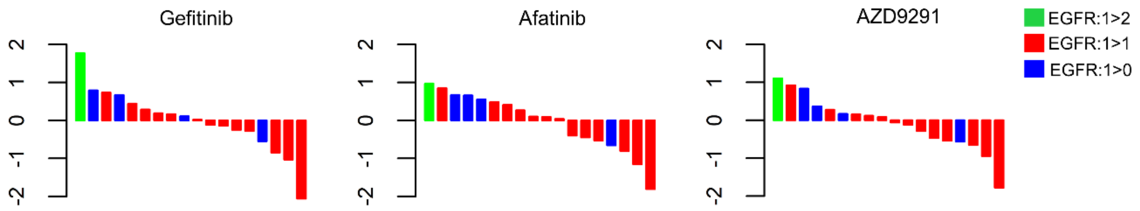

Supplementary Fig. 1. Three EGFR-TKI responses of an EGFR-expansion model patient.

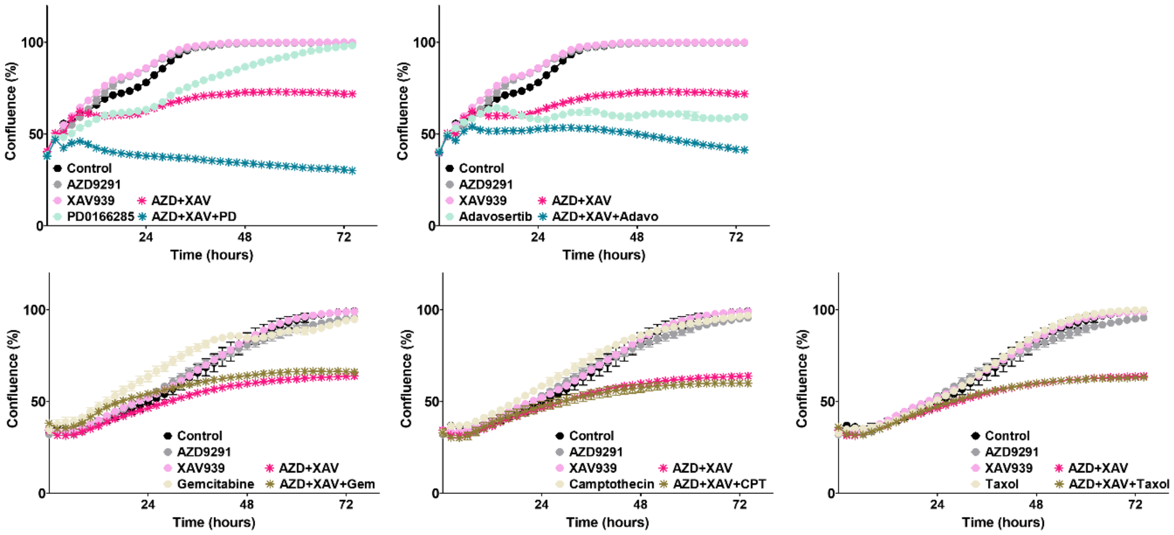

Supplementary Fig. 2. The synergy investigation of combination therapies of seven drug candidates to target *MYC*+ cells induced EGFR-TKI resistance
